# Evidence and Future Directions for Pediatric Health Care Chatbots: Systematic Review

**DOI:** 10.2196/98432

**Published:** 2026-09-25

**Authors:** Seongwoo Yang, Ju Hyun Jin, Seng Chan You, Min Jung Kim, Kyung Won Kim

**Affiliations:** 1 Yonsei Institute for Digital Health, Yonsei University, Seoul, Republic of Korea; 2 Department of Biomedical Informatics, Yonsei University College of Medicine, Seoul, Republic of Korea; 3 Department of Medicine, Yonsei University College of Medicine, Seoul, Republic of Korea; 4 Department of Pediatrics, National Health Insurance Service Ilsan Hospital, Goyang, Republic of Korea; 5 Department of Pediatrics, Yonsei University Yongin Severance Hospital, Yongin, Republic of Korea; 6 Department of Pediatrics, Yonsei University College of Medicine, 50-1 Yonsei-ro, Seodaemun-gu, Seoul, 03722, Republic of Korea, +82-2-2228-2050

**Keywords:** pediatrics, health care chatbots, AI, digital health, health-related outcomes

## Abstract

**Background:**

Pediatric health care requires distinct considerations, including caregiver involvement and developmental differences in cognition and communication as children gain autonomy, particularly as pediatric health care chatbots gradually emerge. Because childhood and adolescence are formative periods for health behaviors and self-management practices, pediatric chatbots also warrant evaluation against long-term rather than immediate outcomes.

**Objective:**

This study aimed to characterize and synthesize the available evidence on pediatric health care chatbots evaluated for health-related outcomes. Furthermore, by identifying gaps in the existing literature, we sought to propose specific considerations for the design, evaluation, and implementation of pediatric health care chatbots.

**Methods:**

PubMed, Embase, Scopus, PsycINFO, the Cochrane Library, and the Web of Science were systematically searched without publication year restrictions. Randomized controlled trials, mixed methods, and observational studies that evaluated health care chatbots for children (aged <19 y) or caregivers and assessed health-related outcomes were included. Nonoriginal papers, end-of-life or palliative care studies, and non-English publications were excluded. Study quality was assessed using the Mixed Methods Appraisal Tool and the Oxford Levels of Evidence 2.

**Results:**

A total of 9 studies were included, with 5 (55.6%) involving pediatric participants only, while 4 (44.4%) involved caregivers. Six (66.7%) studies lacked a comparator, and only 3 (33.3%) chatbots were AI-based. Health and psychosocial outcomes were mixed, often showing null findings in objective clinical metrics despite some subjective improvements. Behavioral and cognitive outcomes generally showed favorable changes but relied heavily on subjective evaluations. Although chatbots demonstrated explicit developmental tailoring, with designs shifting from caregiver-mediated approaches in early childhood to autonomous, privacy-focused platforms for adolescents, definitive conclusions regarding their robust associations with health-related outcomes cannot be drawn. This is primarily due to pervasive methodological limitations, including the lack of active comparator groups, reliance on short-term metrics, and significant study heterogeneity.

**Conclusions:**

Pediatric health care chatbots are emerging across diverse health care contexts, but the current evidence remains limited and heterogeneous. This review identified developmentally relevant considerations, including caregiver involvement, age-appropriate communication, and developmental differences, that may warrant explicit attention in future chatbot design, evaluation, and implementation.

## Introduction

Health care chatbots, conversational systems that interact with users through natural language, have been increasingly developed and introduced across health care settings [1-3]. These systems encompass rule-based, retrieval-based, and AI-based approaches, including more recent large language model–based applications [4,5]. In adult populations, a substantial number of trials have evaluated chatbot support for preventive behaviors and the self-management of chronic diseases [2,6-12]. The number of pediatric health care chatbots also appears to be gradually increasing [13,14]; nevertheless, pediatric applications remain substantially less common than those developed for adults [2]. Pediatric health care chatbots may offer accessible support for children and caregivers across health education, preventive care, symptom management, and communication with health care services [2,4].

Developing pediatric health care chatbots requires specific considerations because pediatric care differs fundamentally from adult medicine. First, children depend on caregivers, and their health care decisions and health outcomes are strongly influenced by parental understanding and beliefs [15-17]. Accordingly, effective pediatric health care chatbots may need to engage both children and caregivers. Second, cognition develops from concrete thinking in early childhood to abstract reasoning in adolescence [18,19], so a one-size-fits-all chatbot design is unlikely to meet the needs of children across developmental stages [20]. Third, childhood is a formative period in which health behaviors and self-management practices are established [21,22]. Collectively, these considerations limit the direct application of adult-focused chatbot research and call for a pediatric-specific synthesis of how these tools are designed, applied, and experienced by children and caregivers.

Martinengo et al [23] proposed the conceptual framework for health care conversational agents (CHAT), a comprehensive guide addressing key considerations across the design, development, evaluation, and implementation stages of conversational agents. CHAT was informed by interviews with multidisciplinary experts and encompasses a broad range of considerations, including ethics, user involvement, and data privacy. Although CHAT provides valuable general guidance, it does not specifically address developmental differences, child-caregiver dynamics, or age-appropriate communication and safety considerations in pediatric health care. Moreover, the available pediatric evidence remains fragmented across health conditions, chatbot functions, and outcome measures, limiting its translation into clear guidance for development and implementation.

This systematic review aimed to characterize and synthesize the available evidence on pediatric health care chatbots evaluated for health-related outcomes. Furthermore, by identifying gaps in the existing literature, we sought to propose specific considerations for the design, evaluation, and implementation of pediatric health care chatbots.

## Methods

### Search Strategies

This review systematically searched PubMed, Embase, Scopus, PsycINFO, the Cochrane Library, and the Web of Science without publication year restrictions, in accordance with PRISMA (Preferred Reporting Items for Systematic Reviews and Meta-Analyses) 2020 guidelines [24,25]. The protocol was registered with the International Prospective Register of Systematic Reviews (PROSPERO ID: CRD420251183243). The search combined controlled vocabulary (eg, MeSH terms) and free-text keywords related to AI, chatbots or conversational agents, and pediatric populations or caregivers, without specifying comparator or outcome terms (Multimedia Appendix 1). To maximize search sensitivity in an emerging and inconsistently indexed field, our search strings incorporated broad overarching terms alongside specific controlled vocabularies.

### Study Selection

After deduplication, 2 reviewers (SY and JHJ) independently screened titles and abstracts. Full texts of the screened studies were assessed for inclusion. Eligible studies were original peer-reviewed evaluations of health care chatbots for children (aged <19 y) or their caregivers, including randomized controlled trials (RCTs), nonrandomized quantitative studies, mixed methods studies, and qualitative studies. In addition, to distinguish this review from purely technical literature, inclusion strictly required the evaluation of health-related outcomes using fully operational chatbots. Nonoriginal papers (eg, reviews, editorials, or study protocols), non-English publications, end-of-life or palliative care studies, those merely presenting conceptual frameworks or early-stage prototypes, and studies reporting solely on nonclinical outcomes (eg, technical feasibility, usability, or acceptability without health-related outcomes) were excluded. Reasons for full-text exclusion were documented (Multimedia Appendix 2), and disagreements were resolved through discussion.

### Data Extraction

Data extraction was performed using a standardized extraction framework developed a priori. For each included study, the following information was extracted: author, year of publication, country, study design, target population, sample size, types of outcomes, chatbot characteristics, mechanism (rule-based, retrieval-based, or generative AI), comparator, and reported outcomes. For qualitative and mixed methods studies, key themes and supporting findings related to user and caregiver experience, acceptability, and implementation were extracted. Extraction was performed by one reviewer and verified by a second.

### Data Synthesis and Analysis

Owing to the heterogeneity in primary outcomes, chatbot functions, outcome measures, and study designs, a meta-analysis was not feasible. Therefore, a structured narrative synthesis was performed, in which quantitative findings and qualitative themes were synthesized within a common framework organized by our research questions. Reported health-related outcomes were categorized by outcome domain, encompassing health and psychological, or behavioral and cognitive outcomes, from a structured taxonomy [26]. Within each domain, quantitative results were summarized by direction and magnitude where allowed, and qualitative findings were juxtaposed with the corresponding quantitative results to assess convergence or divergence. In addition, chatbot content and design features were also descriptively compared across developmental stages.

### Quality Assessment

Study quality was evaluated with the Mixed Methods Appraisal Tool (MMAT) [27,28], which was selected because the included studies comprised heterogeneous designs, including randomized, nonrandomized, and mixed methods studies, and the MMAT permits consistent appraisal across all of these designs. Two authors (SY and JHJ) independently performed the assessments and subsequently compared evaluations. Discrepancies were resolved through discussion until consensus was reached. The level of evidence of each study was additionally classified using the Oxford Levels of Evidence 2 [29].

## Results

### Study Characteristics

**Figure 1.**
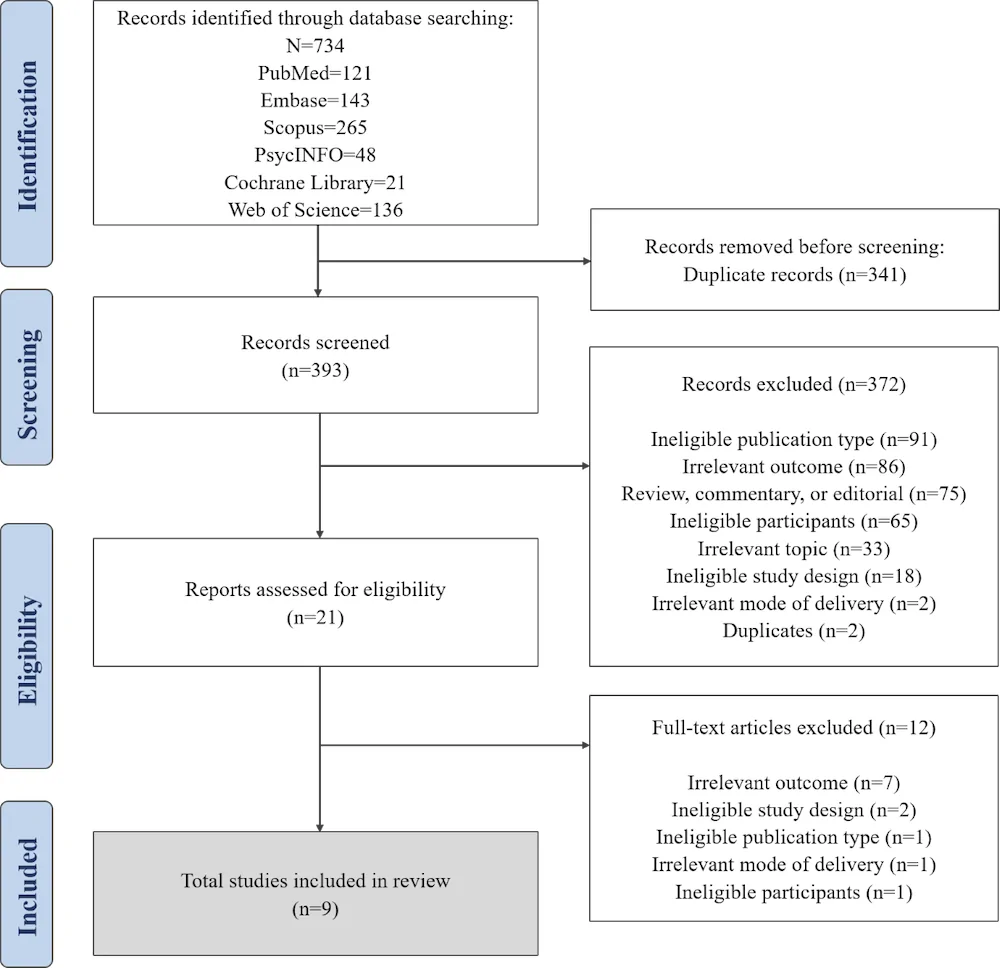
Preferred Reporting Items for Systematic Reviews and Meta-Analyses (PRISMA) flow diagram.

Across the selected databases, 734 studies were identified, of which 393 remained after removing duplicates. We primarily excluded studies reporting irrelevant outcomes, such as mere feasibility. Title and abstract screening yielded 21 studies for full-text review, with strong interrater agreement between the 2 reviewers (SY and JHJ) at both the initial screening (Cohen κ=0.75) and full-text review (Cohen κ=0.81) stages [30]. Ultimately, 9 studies were included in the qualitative synthesis (Figure 1) according to the PRISMA guidelines (Checklist 1). The characteristics of the included studies are presented in Table 1. Of the 9 studies, 5 (55.6%) involved pediatric participants only, 3 (33.3%) involved caregivers only, and 1 (11.1%) involved both pediatric participants and caregivers. Mixed methods designs were the most common, accounting for 5 (55.6%) studies. Most studies lacked a comparator group (n=6, 66.7%), and no standalone qualitative studies meeting the criteria were identified.

**Table 1.** Characteristics of pediatric health care chatbots in the included studies (N=9).

Section/variable/category	Studies, n (%)
Study characteristics	
Country	
United States	2 (22.2)
Thailand	2 (22.2)
UK	1 (11.1)
China	1 (11.1)
South Korea	1 (11.1)
New Zealand	1 (11.1)
Brazil	1 (11.1)
Study design	
Mixed methods study	5 (55.6)
Randomized controlled trial	2 (22.2)
Longitudinal observational study	1 (11.1)
Exploratory pilot study	1 (11.1)
Control condition	
Usual care	2 (22.2)
Active comparator	1 (11.1)
No comparator	6 (66.7)
Participant configuration	
Pediatric participants only	5 (55.6)
Caregivers only	3 (33.3)
Pediatric participants and caregivers	1 (11.1)
Health and psychological^a^^,^^b^	
Physical functioning	4 (44.4)
Emotional functioning or well-being	3 (33.3)
Psychiatric outcomes	2 (22.2)
Endocrine outcomes	1 (11.1)
General outcomes	1 (11.1)
Behavioral and cognitive^a^	
Cognitive functioning	5 (55.6)
Delivery of care	3 (33.3)
Need for further intervention	2 (22.2)
Social functioning	1 (11.1)
Chatbot characteristics	
Technical approach	
Rule-based	4 (44.4)
AI-based	3 (33.3)
Other or not clearly reported	2 (22.2)
Delivery channel	
Social media platform	5 (55.6)
Standalone mobile application	3 (33.3)
Web-based or hospital digital platform	1 (11.1)
Chatbot function^b^	
Health information and education	5 (55.6)
Behavioral feedback and self-monitoring	3 (33.3)
Emotional support and empathic interaction	2 (22.2)

a Health-related outcome.

b Categories are not mutually exclusive, as some studies reported more than one health-related outcome or chatbot function.

Most chatbot interventions were delivered via social media platforms (n=5, 55.6%), and 7 of 9 (77.8%) did not incorporate a visual avatar or persona. Health information and education were the most common chatbot functions (n=5, 55.6%), followed by behavioral feedback and self-monitoring (n=3, 33.3%) and emotional support and empathic interaction (n=2, 22.2%). Regarding technical approaches, 4 (44.4%) chatbots were rule-based [31-37], 3 (33.3%) were AI-based [31,33-37], and 2 (22.2%) used other or unclearly reported approaches [38,39]. Regarding health-related outcomes, the most commonly assessed domains were cognitive functioning (n=5, 55.6%), physical functioning (n=4, 44.4%), and emotional functioning or well-being (n=3, 33.3%). Fewer studies assessed delivery of care (n=3, 33.3%), psychiatric outcomes (n=2, 22.2%), or need for further intervention (n=2, 22.2%).

### Health-Related Outcomes in Pediatric Health Care Chatbots

The health-related outcomes evaluated across the 9 included studies were the primary basis for our synthesis, categorized specifically into health and psychosocial outcomes and behavioral and cognitive outcomes, alongside essential context regarding the study populations, assessment timing, and key clinical findings (Table 2).

**Table 2.** Health-related outcomes and key findings of pediatric health care chatbot studies.

First author, year (country)	Pediatric population (sample size)	Caregiver participation	Pediatric age	Caregiver age	Health-related outcomes	Health and psychosocial outcomes	Behavioral and cognitive outcomes	Assessment timing	Key findings
Bray [38], 2020 (UK)	Children undergoing a medical procedure (n=80)	Children and parents	Intervention: 12 y; control: 10.5 y	NR^a^	Procedure preparation	Procedural anxiety in children and parents	Procedural knowledge, satisfaction, and involvement	Baseline, immediately before the procedure, and within 10 min after the procedure	Preprocedure anxiety was lower in the chatbot group among children (*P*=.008) and parents (*P*=.05).
Vertsberger [37], 2022 (United States)	Adolescents (n=10,387)	None	14‐18 y	—^b^	General well-being	Well-being	—	Baseline and every 6 weeks; 2‐5 assessments	Mean WHO-5^c^ score increased from 39.28 at baseline to 53.64 at follow-up (*P*<.001).
Escobar-Viera [32], 2023 (United States)	Rural LGBTQ+^d^ adolescents with depression and social isolation (n=20)	None	16.6 (1.5) y	—	Depression and social isolation	Depressive symptoms; social isolation	—	Baseline and 1 week	No significant changes were observed in depressive symptoms or social isolation.
Massa [35], 2023 (Brazil)	Adolescent men who have sex with men (n=130)	None	15‐19 y	—	HIV prevention and PrEP^e^ demand creation	—	PrEP clinic scheduling and PrEP uptake	NR	PrEP clinic scheduling and uptake were lower than those reported for other social network–based demand-creation strategies
Lee [39], 2024 (South Korea)	Adolescents (n=42)	None	15.0 (0.7) y	—	Reduction of sugar-sweetened beverage consumption	—	Beverage-related knowledge and beverage intake	Baseline, daily during the 2-week intervention, and postintervention	Weekly sugar intake decreased by approximately 60% (*P*=.03), and beverage-related knowledge improved significantly.
Hunsrisakhun [33], 2024 (Thailand)	Young children (n=303)	Caregivers only	23.4 (9.9) mo	32.4 (7.4) y	Early childhood caries prevention	Dental caries; dental plaque	Oral health knowledge, preventive behaviors, and perceptions	Baseline, 3 months, and 6 months	No significant between-group differences were observed in dental caries or plaque outcomes; oral health knowledge and preventive behaviors improved within groups.
Hou [36], 2025 (China)	Adolescent girls eligible for HPV^f^ vaccination (n=2,671)	Caregivers only	13.1 (1.1) y	40.4 (4.6) y	HPV vaccination	Vaccine confidence	Verified or scheduled HPV vaccination, vaccination consultation, and vaccine literacy	Baseline and 2 weeks	Verified HPV vaccination or scheduled appointment was higher in the chatbot group (RR^g^ 3.85, 95% CI 2.48‐5.97); vaccination consultation was also higher (RR 2.73).
Pupong [34], 2025 (Thailand)	Young children aged 6‐36 months (n=58)	Caregivers only	20.9 (7.9) mo	34.5 (8.6) y	Early childhood oral health promotion	—	Toothbrushing behavior and PMT^h^-related perceptions	Baseline and 2 months	Reported toothbrushing increased from 72.4% to 93.1%, and the mean PMT score increased by 0.5 points.
Boggiss [31], 2025 (New Zealand)	Adolescents with T1DM^i^ (n=40)	None	14.18 (1.11) y	—	Diabetes self-management	HbA_1c_^j^, diabetes distress, resilience, stress, self-efficacy, self-compassion, and emotional well-being	Self-care behaviors	Baseline, 6 weeks, and 12 weeks	At 6 weeks, diabetes distress decreased (reported mean difference −4.71) and emotional well-being increased (+1.41); HbA_1c_ remained stable.

a NR: not reported.

b Not applicable.

c WHO-5: World Health Organization-Five Well-Being Index.

d LGBTQ+: lesbian, gay, bisexual, transgender, queer or questioning, and other sexual and gender identities.

e PrEP: pre-exposure prophylaxis.

f HPV: human papillomavirus.

g RR: risk ratio.

h PMT: protection motivation theory.

i T1DM: type 1 diabetes mellitus.

j HbA_1c_: glycated hemoglobin.

Regarding health and psychological outcomes, the included studies reported mixed findings. While some interventions were associated with lower preprocedural anxiety among children and parents [38], decreased diabetes-related distress among adolescents with type 1 diabetes [31], and improved overall well-being scores in a longitudinal observational study [37], studies assessing objective clinical or psychiatric outcomes reported null findings. Although the chatbot intervention for early childhood caries improved behavioral knowledge, a randomized trial revealed no significant differences in objective dental caries or plaque indices between the intervention and in-person training group [33]. Similarly, while a chatbot was associated with improved emotional well-being in adolescents with type 1 diabetes, physiological markers such as HbA_1c_ (glycated hemoglobin) remained stable with no significant changes [31]. Furthermore, an exploratory pilot study of rural LGBTQ+ (lesbian, gay, bisexual, transgender, queer or questioning, and other sexual and gender identities) adolescents found no significant improvement in depressive symptoms or social isolation [32].

Regarding behavioral and cognitive outcomes, studies reported positive changes, though these were primarily based on subjective or caregiver-reported metrics without long-term objective validation. For instance, rule-based chatbots targeting early childhood oral health improved caregiver-reported toothbrushing behaviors [34] and oral health knowledge over time [33]. Similarly, a chatbot addressing adolescent beverage consumption was associated with a 60% reduction in weekly sugar intake and improved dietary knowledge [39]. On the other hand, studies evaluating health care use outcomes yielded divergent results. A cluster RCT assessing an AI-driven chatbot for human papillomavirus (HPV) vaccination reported a higher rate of verified or scheduled vaccination appointments (risk ratio 3.85) and vaccination consultations compared with usual care [36]. Conversely, an AI-powered chatbot designed for PrEP demand creation among adolescent men who have sex with men resulted in lower clinical scheduling and PrEP uptake compared with other peer-led social network strategies [35].

### Pediatric Health Care Chatbots Across Developmental Stages

Distinct patterns in chatbot content and features were observed across developmental stages (Table 3). In early childhood (0‐3 y), chatbot interventions were primarily caregiver-mediated and focused on health education and caregiver behavior change [33,34]. These interventions commonly incorporated simple language and multimodal content, including illustrations, infographics, animations, songs, audio, and video, with some incorporating personalized conversational features such as remembering the child’s name and revisiting previous discussions. In childhood (4‐12 y), chatbot features placed greater emphasis on direct child engagement and interactive learning, even when delivered for shared family use [38]. While parents reported using the tool together with their child to prepare for an upcoming procedure, the platforms maintained deeply child-centered elements. They incorporated tailored information, avatars, gameplay, augmented reality, and self-paced learning, while also supporting communication between children and their caregivers. In adolescence (13‐19 y), chatbot applications addressed a broader range of topics, including health behavior modification, mental health and well-being, chronic disease self-management, vaccination, and sexual health [31,32,35-37,39]. Aside from 1 HPV vaccination chatbot targeted at parents rather than adolescents, these interventions were used directly by the adolescents themselves. Features commonly emphasized conversational and age-appropriate communication, personalization, reminders, and accessibility. Several interventions incorporated adolescent-oriented language, emojis, or cultural and identity-relevant expressions, although limitations related to unnatural or repetitive conversations were also reported. Privacy and safety considerations were more prominently reported in adolescent-focused interventions, particularly those addressing mental health or sensitive health topics.

**Table 3.** Characteristics of pediatric health care chatbots across developmental stages.

Feature	Early childhood (0‐3 y)	Childhood (4‐12 y)	Adolescence (13‐19 y)
Primary user	Predominantly caregiver	Child with caregiver support	Predominantly adolescent
Caregiver role	Central; intervention mediated through caregiver	Supportive and dyadic	Limited; condition-dependent
Content focus	Health education and caregiver behavior change	Health education and preparation	Prevention, self-management, psychosocial, and sensitive health issues
Communication	Simple, clear, and caregiver-oriented	Child-centered and developmentally tailored	Informal, conversational, age-sensitive, and identity-sensitive
Multimedia	Images, animation, songs, audio or video, and games	Avatar, gameplay, augmented reality, and visual information	Text/chat, infographics, video, emojis or memes, and branching content
Personalization	Child name and tailored dialogue	Customized avatar and tailored information	Behavioral tailoring, persona, conversational memory, and personalized support
Engagement strategies	Repetition and daily brief exposure	Interactive and self-paced learning	Reminders, conversational engagement, and 24/7 accessibility
Privacy and safety	Limited reporting	Limited reporting	Reporting particularly for mental health and sensitive topics (eg, a waiver of parental consent)
Developmental emphasis	Caregiver-mediated action	Comprehension and increasing autonomy	Autonomy, privacy, identity, and sustained self-management

### Quality Assessment

The overall study quality varied across designs, with substantial interrater agreement between the 2 reviewers (SY and JHJ; Cohen κ=0.61; Table 4) [30]. Based on the domain-level MMAT assessment, qualitative components generally demonstrated high methodological quality; however, nonrandomized studies revealed critical gaps, particularly in population representativeness (0%) and confounder adjustment (0%). Similarly, the RCTs showed limitations in blinding, baseline comparability, and outcome completion. Mixed methods studies predominantly struggled with the effective integration of components and the resolution of divergences between quantitative and qualitative results. These domain-specific findings indicate considerable methodological variability across the included studies and underscore the need for the cautious interpretation of the synthesized evidence. Detailed assessments by the MMAT and the Oxford Center for Evidence-Based Medicine criteria are provided in Multimedia Appendix 3.

**Table 4.** Quality assessment summary of the included studies (N=9).

Criteria for quality assessment	MMAT^a^ quality rating, n (%)
Qualitative (n=5)	
Appropriate answer to the research question	5 (100)
Adequate data collection	4 (80)
Adequate findings from the data	4 (80)
Verified interpretation	5 (100)
Coherence	5 (100)
Nonrandomized studies (n=7)	
Representative population	0 (0)
Exposure or outcome measurement	7 (100)
Completion of outcome data	6 (86)
Adjustment of confounders	0 (0)
Intervention or exposure as intended	6 (86)
RCTs^b^ (n=2)	
Appropriate randomization	2 (100)
Comparable groups at baseline	1 (50)
Completion of outcome data	1 (50)
Blinding of assessors	1 (50)
Adherence to the intervention	1 (50)
Mixed methods studies^c^ (n=5)	
Adequate rationale	5 (100)
Effective integration of different components	1 (20)
Adequate interpretation	1 (20)
Divergences and inconsistencies resolved	0 (0)
Adherence to the quality criteria of each method	1 (20)

a MMAT: Mixed Methods Appraisal Tool.

b RCT: randomized controlled trial.

c Mixed methods studies have also been assessed by items for qualitative and nonrandomized studies.

## Discussion

### Principal Findings

This systematic review synthesizes the current evidence from 9 studies evaluating health care chatbots for pediatric patients and their caregivers. Overall, our synthesis indicates that while these tools show preliminary utility, the field remains in a nascent stage of clinical validation. Specifically, the included studies addressed a diverse range of health domains, including general well-being, procedural preparation to reduce anxiety, reduction of sugar intake from beverages, toothbrushing education and caries prevention, and encouraging medical resource use. However, the quality of evidence was generally low, and the direction of effects was not consistent across the studies. Taken together, the impact of chatbots on health-related outcomes remains inconclusive. Nevertheless, the included studies point to several critical considerations for future chatbot design and evaluation: age-appropriate design and context, caregiver involvement, long-term outcome assessment, and safety and privacy issues.

Several factors likely explain this lack of consistent evidence. First, this review included a relatively small number of studies, in part because of its rigorous scope: only studies reporting health-related outcomes (clinical or patient-reported) were eligible, whereas those limited merely to feasibility, usability, or engagement fell outside this scope. Additionally, unlike previous reviews [40,41], the strict age criterion (<19 y) excluded research that grouped adolescents with young adults. Second, the strength of the underlying literature is constrained by study design. Only 2 of the 9 included studies used an RCT design, while the majority used a single-group or mixed methods design lacking an active comparator. This methodological gap aligns with a broader pattern in pediatric research, where therapeutic device and digital health development are often hindered by small sample sizes and short follow-up periods due to inherent ethical, regulatory, and financial barriers [42,43].

This study showed that pediatric care needs to consider a dynamic trajectory of cognitive and emotional development, necessitating approaches that extend beyond uniform adult-centered models [44]. Developing interventions for pediatric populations therefore requires deliberate attention to age-appropriate design and delivery methods. Our review reflects this pattern, highlighting that the most effective chatbots were tailored to specific developmental stages. For younger children, interventions incorporated gamification and augmented reality–based avatars to reduce preprocedural anxiety [38]. In contrast, adolescent-focused platforms adopted a peer-like, emoji-based persona to foster engagement in dietary self-monitoring [39]. This digital tailoring directly mirrors established clinical practice, in which tools and services offered to pediatric populations, such as pain assessment and procedural preparation, are already tailored to a child’s developmental level [43,45-47]. Consequently, pediatric health care chatbots should not represent a simplified adaptation of adult software but rather a developmentally tailored interface that reflects the evolving cognitive capacities and social needs of children and adolescents.

Caregiver involvement in the included chatbots was shaped primarily by the child’s developmental stage and, for certain health decisions, by parental decision-making authority. For infants and toddlers, caregivers were the sole practical users, since children at this age could not yet interact with a chatbot directly [33,34]. As children grew older, adolescent-focused chatbots were typically used directly by adolescents. One exception was the HPV vaccination chatbot, where mothers made up the majority of users, since vaccination decisions require parental consent [36]. Caregiver involvement nonetheless remains valuable through 2 distinct pathways. First, chatbots that build caregivers’ health literacy can improve children’s health outcomes even without directly engaging the child. Second, when children and caregivers use a chatbot together, this shared use can open family discussions and reinforce health behaviors [48]. These findings suggest that developers of pediatric chatbots should explicitly define the target age group and decide, based on the child’s developmental stage and the health topic, whether and how caregivers should be involved. However, robust clinical evidence is still required, as the pediatric evidence base lags behind that for adults, in which a substantial number of trials have accumulated.

On the other hand, for topics such as sexual or mental health, caregiver involvement can invade an adolescent’s privacy; chatbots addressing these topics therefore target adolescents alone [31,32,35]. This aligns with established adolescent health care principles, which recognize age-appropriate involvement of caregivers while emphasizing the need to protect adolescents’ privacy and autonomy, especially when addressing sensitive topics [49]. Beyond privacy, ensuring equitable access remains a critical challenge [50]. Although some studies specifically targeted underserved populations [31,32], others revealed disparities in chatbot uptake and engagement by education level and ethnicity, generally favoring more advantaged subgroups [35,39]. Accordingly, future implementations must prioritize inclusive design strategies to prevent digital health interventions from inadvertently widening existing health disparities.

Pediatric evidence is often constrained by small sample sizes and short follow-up periods, as shown in previous studies [51-53], a pattern also reflected in this review, where the longest follow-up period extended to at most 6 months. Because health behaviors established in childhood significantly influence adult chronic disease risk [54-56], the current short-term focus necessitates long-term and adequately powered studies to verify sustained benefits [57]. The combination of a smaller market and weaker regulatory and financial incentives for pediatric-specific research helps explain why longitudinal evidence remains slow to accumulate [42,43]. To address this gap, diversified public funding and real-world evidence generation could offer a path forward [42].

### Considerations for Pediatric Health Care Chatbots

**Figure 2.**
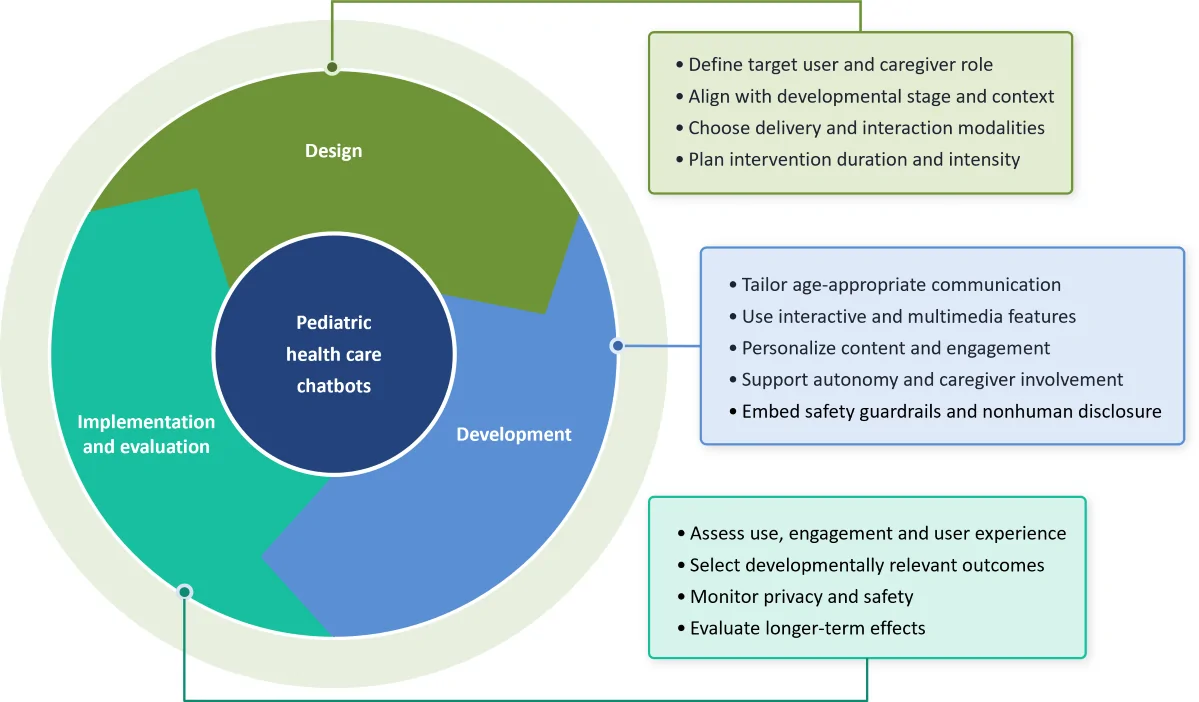
Considerations for health care chatbots in pediatric care based on the conceptual framework for health care conversational agents (CHAT).

Building upon the identified evidence gaps and recurring themes, we outline several pediatric-specific considerations that extend general-purpose frameworks like CHAT [23], organizing these recommendations according to its core domains (Figure 2). The considerations are identified from the findings of the included studies and are intended to reflect the currently available evidence rather than an empirically validated framework. Pediatric chatbot design may begin by defining the target users based on developmental stage and relevant health topics. Interventions for infants and toddlers practically target caregivers [16,33,34], whereas those addressing sensitive topics target adolescents directly to protect privacy [31,32,35]. This pattern is not strictly linear: caregiver involvement can also increase in specific contexts or around key decisions, such as parental consent for vaccination [36] or parental coparticipation in procedural preparation [38]. Delivery modalities, as well as intervention duration and intensity, should align with the developmental context, incorporating gamification for younger children [38] and developmentally appropriate language for adolescents [35,39]. Technologically, the rigidity of current rule-based systems highlights the need for more adaptive models capable of interpreting and responding to pediatric users [31-34,37].

Pediatric implementation also demands specific safety and privacy protocols. Because children may readily trust friendly or empathetic agents [40], systems must embed safeguards, such as clear nonhuman disclosures and human-in-the-loop escalation pathways [31,37]. Privacy mechanisms must balance parental consent with adolescent autonomy, particularly for sensitive interventions requiring independent consent [32,35]. The limited reporting of AI-specific implementation details also precluded assessment of how individual AI components contributed to the reported outcomes. Moving forward, transparently reporting specific AI implementations (eg, model type, prompt design, and retrieval-augmented generation) in accordance with emerging guidelines [58,59] is required to ensure informational autonomy. Combining this technical transparency with large-scale, longitudinal trials that rigorously evaluate usability, user engagement, and adverse events is necessary to establish these conversational agents as safe and evidence-based pediatric interventions [21,60] with endpoint indicators of health prognosis.

### Strengths and Limitations

This review presents 3 main strengths. First, it focuses specifically on pediatric populations, analyzing distinct interaction models such as caregiver-mediated interventions and developmental vulnerabilities. Second, the study uses the CHAT framework to systematically categorize the evidence, identifying pediatric-specific requirements regarding clinical safety and privacy. Third, it extends beyond a descriptive mapping of current tools to evaluate clinical and behavioral outcomes, methodological limitations (eg, inconsistent technical reporting and lack of safety surveillance), and digital health equity gaps.

This study also has several limitations. First, the relatively small number of included studies and their substantial heterogeneity regarding study design, sample size, health care context, outcomes, and types of intervention precluded quantitative meta-analysis, making narrative synthesis more appropriate. This heterogeneity, together with the frequent lack of comparator groups and inconsistent assessment of objective clinical outcomes, limits the credibility and generalizability of the findings and warrants cautious interpretation of effectiveness. Also, we could not statistically evaluate publication bias or formally assess the overall certainty of evidence. Second, our eligibility criteria regarding chatbot maturity and outcome measures limited the scope of included studies by excluding early-stage research focused primarily on usage, feasibility, or usability without health-related or behavioral outcomes. Furthermore, although the broader term “pediatric health care chatbots” was adopted, the original search strategy was primarily framed around medical AI chatbots, which may have missed studies indexed under broader or non-AI terminology. Third, the short follow-up periods (up to 6 mo) limit assessment of the sustainability of behavioral changes, particularly as habit formation has been reported to vary widely, from 4 to 335 days [51]. Adequately powered studies, including RCTs where appropriate, with longer follow-up would strengthen the evidence base. Finally, safety and underlying AI technologies were inconsistently reported across studies, limiting their comprehensive assessment, and our proposed framework, derived from the available evidence in this review, currently lacks empirical and longitudinal validation.

### Conclusions

This review highlights several developmentally relevant considerations for pediatric health care chatbots, including caregiver involvement, age-appropriate communication, and differences in cognitive and behavioral needs across developmental stages. Although pediatric health care chatbots are emerging across diverse health care contexts, the current evidence remains limited and heterogeneous, and developmentally relevant considerations were inconsistently addressed across studies. Based on the available evidence, an integrated pediatric-specific approach that aligns developmental stage and caregiver roles across chatbot design, development, implementation, and evaluation may provide useful considerations for future pediatric health care chatbots. In addition, longer-term evaluation of these chatbots is needed to determine whether observed changes are sustained over time.

## Supplementary material

10.2196/98432 Multimedia Appendix 1 Search strategy for the systematic review by database.

10.2196/98432 Multimedia Appendix 2 Studies excluded at full-text review.

10.2196/98432 Multimedia Appendix 3 Detailed quality assessment by the Mixed Methods Appraisal Tool and Oxford Centre for Evidence-Based Medicine.

10.2196/98432 Checklist 1 PRISMA checklist.

## Abbreviations

HbA_1c_: glycated hemoglobin
HPV: human papilloma virus
LGBTQ+: lesbian, gay, bisexual, transgender, queer or questioning, and other sexual and gender identities
MMAT: Mixed Methods Appraisal Tool
PRISMA: Preferred Reporting Items for Systematic Reviews and Meta-Analyses
RCT: randomized controlled trial
