# Supplementary material for: Evidence and Future Directions for Pediatric Health Care Chatbots: Systematic Review
Source: J Med Internet Res. 2026 Sep 25;28:e98432. doi: 10.2196/98432 (PMC13614210; doi:10.2196/98432)
Supplement: Supplementary file 2 [file jmir-v28-e98432-s001.docx]

Multimedia Appendix 1. Search Strategy for the Systematic Review by Database

| Search strategy for PubMed | Search strategy for Embase |
| --- | --- |
| #1. "medicine"[MeSH Terms] OR "medical"[Title/Abstract] OR "medicine"[Title/Abstract] OR "health*"[Title/Abstract]  #2. "generative artificial intelligence"[MeSH Terms] OR "artificial intelligence"[Title/Abstract]  #3. "chatbot*"[Title/Abstract] OR "conversational agent*"[Title/Abstract]  #4. "pediatrics"[MeSH Terms] OR "pediatric*"[Title/Abstract] OR "child*"[Title/Abstract] OR "adolescen*"[Title/Abstract]  #5. #1 AND #2 AND #3 AND #4 | #1. 'health care'/exp OR 'health care' OR medicine:ti,ab OR medical:ti,ab OR health*:ti,ab  #2. 'artificial intelligence'/exp OR 'generative artificial intelligence':ti,ab OR 'artificial intelligence':ti,ab  #3. chatbot*:ti,ab OR 'conversational agent*':ti,ab  #4. 'child'/exp OR pediatric*:ti,ab OR child*:ti,ab OR adolescen*:ti,ab  #5. #1 AND #2 AND #3 AND #4 |
| Search strategy for Scopus | Search strategy for PsycINFO |
| #1. (TITLE-ABS-KEY ( medicine OR medical OR health* ) )  #2. (TITLE-ABS-KEY ("generative artificial intelligence" OR "artificial intelligence" ) )  #3. (TITLE-ABS-KEY ( chatbot* OR "conversational agent*") )  #4. (TITLE-ABS-KEY ( pediatric* OR child* OR adolescen* ) )  #5. #1 AND #2 AND #3 AND #4 | S1. (DE "Health" OR SU "Medical Research" OR medicine OR medical OR health*)  S2. (DE "Artificial Intelligence" OR "generative artificial intelligence" OR "artificial intelligence")  S3. (chatbot* OR "conversational agent*")  S4. (DE "Children and Adolescents" OR pediatric* OR child* OR adolescen*)  S5. S1 AND S2 AND S3 AND S4 |
| Search strategy for the Cochrane Library | Search strategy for Web of Science |
| #1 MeSH descriptor: [Medicine] explode all trees  #2 (medicine OR Medical OR health*):ti,ab,kw  #3 #1 OR #2  #4 MeSH descriptor: [Generative Artificial Intelligence] explode all trees  #5 (generative artificial intelligence OR artificial intelligence*):ti,ab,kw  #6 #4 OR #5  #7 (chatbot* OR conversational agent*):ti,ab,kw  #8 MeSH descriptor: [Pediatrics] explode all trees  #9 (pediatric* OR child* OR adolescen*):ti,ab,kw  #10 #8 OR #9  #11 #3 AND #6 AND #7 AND #10 | #1. TS=(medicine OR medical OR health*)  #2. TS=("generative artificial intelligence" OR "artificial intelligence")  #3. TS=(chatbot* OR "conversational agent*")  #4. TS=(pediatric* OR child* OR adolescen*)  #5. #4 AND #3 AND #2 AND #1 |
