# Supplementary material for: Evidence and Future Directions for Pediatric Health Care Chatbots: Systematic Review
Source: J Med Internet Res. 2026 Sep 25;28:e98432. doi: 10.2196/98432 (PMC13614210; doi:10.2196/98432)
Supplement: Supplementary file 3 [file jmir-v28-e98432-s002.docx]

Multimedia Appendix 2. Studies Excluded at Full-Text Review (n=12)

| Study | Reason for exclusion |
| --- | --- |
| Atturu et al. 2024 | Abstract only |
| Berrezueta‑Guzman et al. 2025 | Irrelevant outcome |
| Bjerregaard et al. 2024 | Irrelevant intervention |
| Crutzen et al. 2011 | Irrelevant outcome |
| Davis et al. 2025 | Irrelevant outcome |
| Ko et al. 2025 | Ineligible subjects |
| Motegi et al. 2025 | Irrelevant outcome |
| Fujita et al. 2025 | Ineligible study design (Withdrawal) |
| Kim et al. 2024 | Irrelevant outcome |
| Beilharz et al. 2021 | Irrelevant outcome |
| Boggiss et al. 2023 | Ineligible study design |
| Lau et al. 2025 | Irrelevant outcome |
