# Supplementary material for: Evidence and Future Directions for Pediatric Health Care Chatbots: Systematic Review
Source: J Med Internet Res. 2026 Sep 25;28:e98432. doi: 10.2196/98432 (PMC13614210; doi:10.2196/98432)
Supplement: Supplementary file 4 [file jmir-v28-e98432-s003.docx]

Multimedia Appendix 3. Detailed Quality Assessment by the Mixed Methods Appraisal Tool and Oxford Centre for Evidence-Based Medicine

The following screening questions were rated as “Yes” for the included studies: 1) Are there clear research questions? and 2) Do the collected data allow to address the research questions?

|  |  |  | QUALITATIVE STUDIES | | | | |
| --- | --- | --- | --- | --- | --- | --- | --- |
| First author | Year | Citation | 1.1. Is the qualitative approach appropriate to answer the research question? | 1.2. Are the qualitative data collection methods adequate to address the research question? | 1.3. Are the findings adequately derived from the data? | 1.4. Is the interpretation of results sufficiently substantiated by data? | 1.5. Is there coherence between qualitative data sources, collection, analysis and interpretation? |
| Bray | 2020 | Mixed-methods | Yes | Yes | Yes | Yes | Yes |
| Boggis | 2025 | Mixed-methods | Yes | Yes | Yes | Yes | Yes |
| Escobar-Viera | 2023 | Non-randomized |  |  |  |  |  |
| Hou | 2025 | Randomized controlled trial |  |  |  |  |  |
| Hunsrisakhun | 2024 | Randomized controlled trial |  |  |  |  |  |
| Lee | 2024 | Mixed-methods | Yes | No | Yes | Yes | Yes |
| Massa | 2023 | Mixed-methods | Yes | Yes | Can’t tell | Yes | Yes |
| Pupong | 2025 | Mixed-methods | Yes | Yes | Yes | Yes | Yes |
| Vertsberger | 2022 | Non-randomized |  |  |  |  |  |

|  |  |  | RANDOMIZED CONTROLLED TRIALS | | | | |
| --- | --- | --- | --- | --- | --- | --- | --- |
| First author | Year | Citation | 2.1. Is randomization appropriately performed? | 2.2. Are the groups comparable at baseline? | 2.3. Are there complete outcome data? | 2.4. Are outcome assessors blinded to the intervention provided? | 2.5 Did the participants adhere to the assigned intervention? |
| Bray | 2020 | Mixed-methods |  |  |  |  |  |
| Boggis | 2025 | Mixed-methods |  |  |  |  |  |
| Escobar-Viera | 2023 | Non-randomized |  |  |  |  |  |
| Hou | 2025 | Randomized controlled trial | Yes | Can't tell | No | Can't tell | Can't tell |
| Hunsrisakhun | 2024 | Randomized controlled trial | Yes | Yes | Yes | Yes | Yes |
| Lee | 2024 | Mixed-methods |  |  |  |  |  |
| Massa | 2023 | Mixed-methods |  |  |  |  |  |
| Pupong | 2025 | Mixed-methods |  |  |  |  |  |
| Vertsberger | 2022 | Non-randomized |  |  |  |  |  |

|  |  |  | NON-RANDOMIZED STUDIES | | | | |
| --- | --- | --- | --- | --- | --- | --- | --- |
| First author | Year | Citation | 3.1. Are the participants representative of the target population? | 3.2. Are measurements appropriate regarding both the outcome and intervention (or exposure)? | 3.3. Are there complete outcome data? | 3.4. Are the confounders accounted for in the design and analysis? | 3.5. During the study period, is the intervention administered (or exposure occurred) as intended? |
| Bray | 2020 | Mixed-methods | Can't tell | Yes | Yes | No | Can't tell |
| Boggis | 2025 | Mixed-methods | Can't tell | Yes | Yes | No | Yes |
| Escobar-Viera | 2023 | Non-randomized | Can't tell | Yes | Yes | No | Yes |
| Hou | 2025 | Randomized controlled trial |  |  |  |  |  |
| Hunsrisakhun | 2024 | Randomized controlled trial |  |  |  |  |  |
| Lee | 2024 | Mixed-methods | No | Yes | Yes | No | Yes |
| Massa | 2023 | Mixed-methods | Can't tell | Yes | Yes | No | Yes |
| Pupong | 2025 | Mixed-methods | Can't tell | Yes | Yes | No | Yes |
| Vertsberger | 2022 | Non-randomized | No | Yes | No | No | Yes |

|  |  |  | MIXED METHODS STUDIES | | | | |
| --- | --- | --- | --- | --- | --- | --- | --- |
| First author | Year | Citation | 5.1. Is there an adequate rationale for using a mixed methods design to address the research question? | 5.2. Are the different components of the study effectively integrated to answer the research question? | 5.3. Are the outputs of the integration of qualitative and quantitative components adequately interpreted? | 5.4. Are divergences and inconsistencies between quantitative and qualitative results adequately addressed? | 5.5. Do the different components of the study adhere to the quality criteria of each tradition of the methods involved? |
| Bray | 2020 | Mixed-methods | Yes | Can't tell | Can't tell | Can't tell | Can't tell |
| Boggis | 2025 | Mixed-methods | Yes | Can't tell | Yes | No | Can't tell |
| Escobar-Viera | 2023 | Non-randomized |  |  |  |  |  |
| Hou | 2025 | Randomized controlled trial |  |  |  |  |  |
| Hunsrisakhun | 2024 | Randomized controlled trial |  |  |  |  |  |
| Lee | 2024 | Mixed-methods | Yes | Can't tell | Can't tell | No | Can't tell |
| Massa | 2023 | Mixed-methods | Yes | Can't tell | Can't tell | No | Can't tell |
| Pupong | 2025 | Mixed-methods | Yes | Yes | Can't tell | Can't tell | Yes |
| Vertsberger | 2022 | Non-randomized |  |  |  |  |  |

* qualitative component is limited (brief open-ended feedback), not appraised separately

|  |  |  |  | OCEBM |
| --- | --- | --- | --- | --- |
| First author | Year | Citation |  |  |
| Bray | 2020 | Mixed-methods |  | 2b |
| Boggis | 2025 | Mixed-methods |  | 4 |
| Escobar-Viera | 2023 | Non-randomized |  | 4 |
| Hou | 2025 | Randomized controlled trial |  | 1b |
| Hunsrisakhun | 2024 | Randomized controlled trial |  | 1b |
| Lee | 2024 | Mixed-methods |  | 4 |
| Massa | 2023 | Mixed-methods |  | 4 |
| Pupong | 2025 | Mixed-methods |  | 4 |
| Vertsberger | 2022 | Non-randomized |  | 4 |

OCEBM, Oxford Centre for Evidence-Based Medicine; OCEBM classification was based on the quantitative effectiveness design (therapy/prevention)
